# Tuberculosis Variant with Rifampin Resistance Undetectable by Xpert MTB/RIF, Botswana

**DOI:** 10.3201/eid2911.230987

**Published:** 2023-11

**Authors:** Chawangwa Modongo, Ivan Barilar, Qiao Wang, Tuduetso Molefi, Topo Makhondo, Stefan Niemann, Sanghyuk S. Shin

**Affiliations:** Victus Global Botswana Organisation, Gaborone, Botswana (C. Modongo);; Forschungszentrum, Borstel, Germany (I. Barilar, S. Niemann);; University of California, Irvine, California, USA (Q. Wang, S.S. Shin);; University of California, Los Angeles, California, USA (Q. Wang);; Botswana Ministry of Health and Wellness, Gaborone (T. Molefi, T. Makhondo); German Center for Infection Research, Partner Site Hamburg-Lübeck-Borstel-Riems, Borstel, Germany (S. Niemann)

**Keywords:** multidrug-resistant tuberculosis, tuberculosis and other mycobacteria, pre–XDR TB, antimicrobial resistance, whole-genome sequencing, molecular diagnostics, molecular epidemiology, respiratory infections, bacteria, rifampin resistance, Botswana

## Abstract

GeneXpert MTB/RIF, a tool widely used for diagnosing tuberculosis, has limitations for detecting rifampin resistance in certain variants. We report transmission of a pre–extensively drug-resistant variant in Botswana that went undetected by GeneXpert. The public health impact of misdiagnosis emphasizes the need for comprehensive molecular testing to identify resistance and guide treatment.

The GeneXpert (Xpert) MTB/RIF assay (Cepheid, https://www.cepheid.com) has enabled rapid molecular diagnosis of tuberculosis (TB) and identification of resistance to rifampin, a critical first-line TB drug (*1*). Operating with minimal infrastructure in a cartridge-based system, the assay is the primary TB diagnostic method in many countries (*2*). Xpert MTB/RIF detects rifampin resistance by identifying mutations in an 81-bp region of the *rpoB* gene but does not detect resistance-conferring mutations outside that region (*3*,*4*). We report a case of a multidrug-resistant (MDR) *Mycobacterium tuberculosis* complex (MTBC) strain in Botswana with a rifampin resistance–conferring mutation, *rpoB* I491F, not detected by Xpert MTB/RIF (*3**,**4*). Genomic epidemiology suggested that the infection was part of a transmission chain spanning >5 years. 

The patient was a 44-year-old man who, in August 2021, sought treatment at a public health clinic in Botswana for cough, fever, and night sweats. Xpert MTB/RIF detected MTBC, but no rifampin resistance. No additional drug susceptibility testing occurred during diagnosis. The case-patient reported no previous TB history. He had also newly tested positive for HIV at the time of TB diagnosis and had a CD4+ T-cell count of 117 cells/mm^3^, reflective of advanced HIV-associated immunosuppression. The case-patient completed the 6-month rifampin-containing regimen 2HRZ(E)/4HR and concurrently started antiretroviral therapy of tenofovir disoproxil, lamivudine, and dolutegravir. His TB signs and symptoms resolved; sputum smear tests became negative, and by February 2022, treatment was successfully completed (Figure).

**Figure:**
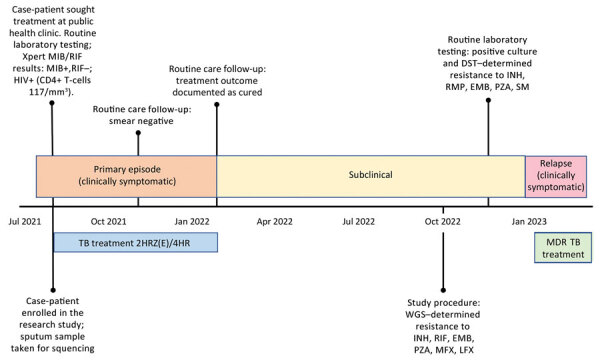
Timeline of events experienced by case-patient in Botswana from study of a rifampin-resistant TB variant not detectable using Xpert MTB/RIF assay (Cepheid, https://www.cepheid.com). Timeline events included routine laboratory procedures, study procedures, and timing of TB treatment. 2HRZ(E)/4HR, standard 6-month tuberculosis treatment regimen (2 months of isoniazid, rifampin and pyrazinamide, with or without ethambutol, followed by 4 months of isoniazid and rifampin); DST, drug susceptibility testing; EMB, ethambutol; INH, isoniazid; LFX, levofloxacin; MDR TB, multidrug-resistant tuberculosis; MFX, moxifloxacin; PZA, pyrazinamide; RMP, rifampin; SM, streptomycin; TB, tuberculosis; WGS, whole-genome sequencing; Xpert, GeneXpert.

At time of TB diagnosis, the patient joined an ongoing genomic TB epidemiology study (https://reporter.nih.gov/project-details/10327709) involving whole-genome sequencing (WGS) of MTBC strains obtained from sputum cultures from TB-diagnosed persons in Botswana study clinics. We performed WGS using Illumina NextSeq 500/2000 (https://www.illumina.com) and analyzed data using MTBseq (https://github.com/ngs-fzb/MTBseq_source), as described elsewhere (*5*). We used variable single-nucleotide polymorphism alignments of MTBC genomes to generate the maximum likelihood phylogeny in IQ-TREE version 1.6.12 (*6*). 

Our October 2022 analysis found that the patient carried a lineage 4.3.3 MTBC strain with the *rpoB* I491F mutation, causing rifampin resistance not detected by Xpert MTB/RIF (*3*,*7*). Moreover, WGS identified the MTBC strain as pre–extensively drug-resistant (pre-XDR), with resistance to isoniazid, ethambutol, pyrazinamide, moxifloxacin, and levofloxacin. Among the 165 study participants enrolled during 2021−2022, no other cases were found for which the isolated genome clustered with the MTBC strain from the case-patient. However, examining MTBC sequences from a previous study (*8*) revealed a clinical MTBC strain, BTB-2087, collected in 2016, in which the genome differed from the present strain, BTC-36, by only 5 single-nucleotide polymorphisms, suggesting that the 2 infections were part of a transmission chain in Botswana that has lasted for >5 years (Appendix Figure). We identified no epidemiologic links between the 2 persons. 

MTBC strains with the *rpoB* I491F mutation have been previously documented in South Africa and Eswatini, and in Eswatini, they constituted >60% of MDR strains (*3*,*4*,*7*). Of interest, the 2 *rpoB* I491F strains in our study belonged to a different MTBC sublineage (4.3.3) than *rpoB* I491F strains previously identified in Eswatini (4.4.1.1) and South Africa (4.1.1.3), pointing toward convergent evolution and selection of strains that escape diagnosis in the region (*3*,*4*). 

A public health investigation conducted in November 2022 found that the case-patient remained asymptomatic but was culture positive for MTBC on *Mycobacteria* growth indicator tube 960 medium (Becton Dickinson; https://www.bd.com). Drug susceptibility testing indicated resistance to isoniazid, rifampin, pyrazinamide, and fluoroquinolones. We tested 5 members of the patient’s household with Xpert MTB/RIF and identified no additional TB cases. In January 2023, the case-patient developed TB symptoms and was placed on an individualized pre–XDR TB treatment of cycloserine, clofazimine, linezolid, bedaquiline, delamanid, pyridoxine, and para-aminosalicyclic acid. No mutations linked to bedaquiline and clofazimine resistance were detected. We are conducting additional investigations to explore the extent of the outbreak of undetected MTBC strains. 

This case demonstrates the clinical and public health utility of whole-genome sequencing for detecting TB drug resistance missed by conventional molecular tests. Of note, failing to detect the patient’s pre–XDR TB resulted in ineffective initial treatment and potentially over a year of infectious TB. Early MDR TB detection could have led to effective initial treatment and reduced the risk of onward transmission. Currently, prevalence of MTBC strains harboring *rpoB* I491F mutation is unknown. Incorporating sequencing into a national TB drug resistance survey and continuing efforts to improve sequencing-based surveillance of drug-resistant TB in Botswana could shed light on the prevalence of MTBC strains harboring the *rpoB* I491F mutation. Those data could be used to inform updates to TB diagnostic guidelines. 

In conclusion, our study highlights the utility of WGS for identifying TB outbreaks and informing public health actions in high–TB burden countries. That approach supports the World Health Organization’s recent strategic guidelines for rapidly communicating results from targeted next-generation sequencing combined with conventional tests to inform TB treatment decisions (*9*). Although Botswana has had remarkable success in improving HIV management, persons not adequately reached by the HIV care system remain at elevated risk of developing TB, including drug-resistant forms (*10*). Additional efforts are needed to ensure that high-quality HIV and TB care are delivered to underserved communities. 

Appendix Additional information about a study of a rifampin-resistant tuberculosis variant not detectable using Xpert MTB/RIF assay, Botswana.
