# Supplementary material for: Tuberculosis Variant with Rifampin Resistance Undetectable by Xpert MTB/RIF, Botswana
Source: Emerg Infect Dis. 2023 Nov;29(11):2403–6. doi: 10.3201/eid2911.230987 (PMC10617350; doi:10.3201/eid2911.230987)
Supplement: Appendix — Additional information about a study of a rifampin-resistant tuberculosis variant not detectable using Xpert MTB/RIF assay, Botswana. [file 23-0987-Techapp-s1.pdf]

Article DOI: https://doi.org/10.3201/eid2911.230987

EID cannot ensure accessibility for supplementary materials supplied by authors. Readers who have difficulty accessing supplementary content should contact the authors for assistance.

Tuberculosis Variant with Rifampicin Resistance Undetectable by Xpert MTB/RIF, Botswana

Appendix

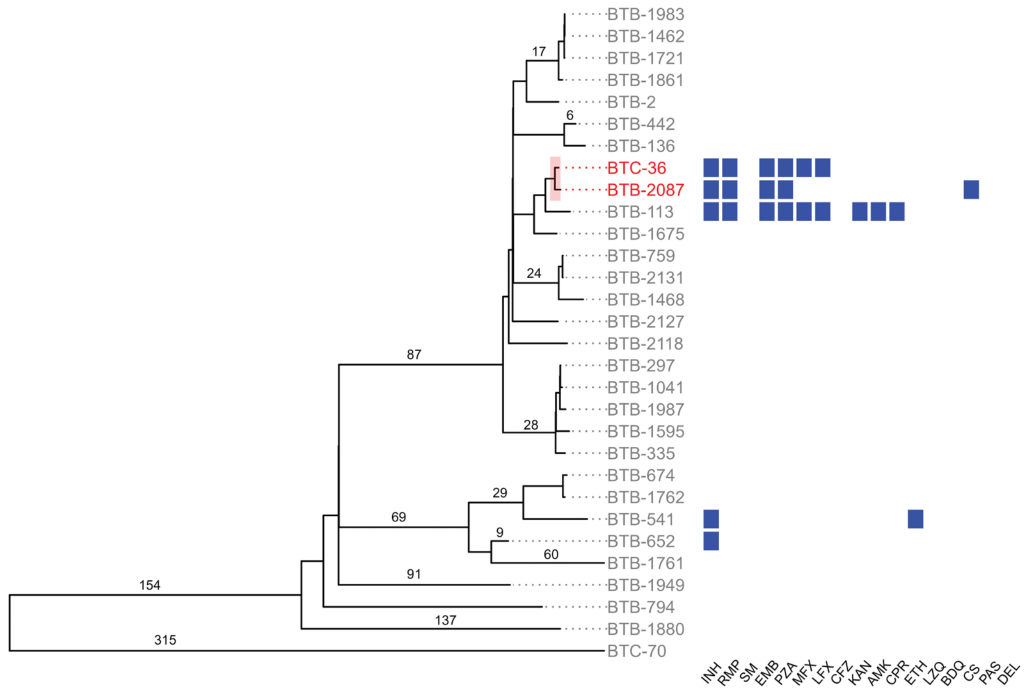

Appendix Figure. Phylogenetic tree of the 2021 sequence (BTC-36) in the context of 2012-2016 TB lineage 4.3.3 isolates, from a study of a rifampicin-resistant tuberculosis variant not detectable using Xpert MTB/RIF assay, Botswana. Clade in red font corresponds to clinical cases harboring the *rpo*B I491F mutation that confers rifampicin resistance. SNP distances between pairs of genomes are labeled on select branches. Blue boxes indicate resistance to antimicrobials listed at bottom of array. INH, isoniazid; RMP, rifampicin; SM, streptomycin; EMB, ethambutol; PZA, pyrazinamide; MFX, moxifloxacin; LFX, levofloxacin; CFZ, clofazimine; KAN, kanamycin; AMK, amikacin; CPR, capreomycin; ETH, ethionamide; LZQ, linezolid; BDQ, bedaquiline; CS, cycloserine; PAS, para-aminosalicylic acid; DEL, delamanid.s
